# Correction: Gvozdenović et al. Impact of Vitamin D Status and Nutrition on the Occurrence of Long Bone Fractures Due to Falls in Elderly Subjects in the Vojvodina Region of Serbia. *Nutrients* 2024, *16*, 2702

**DOI:** 10.3390/nu18071036

**Published:** 2026-03-25

**Authors:** Nemanja Gvozdenović, Ivana Šarac, Andrijana Ćorić, Saša Karan, Stanislava Nikolić, Isidora Ždrale, Jelena Milešević

**Affiliations:** 1 Faculty of Medicine, University of Novi Sad, 21137 Novi Sad, Serbia; nemanja.gvozdenovic@mf.uns.ac.rs (N.G.); 912007d23@mf.uns.ac.rs (A.Ć.); sasa.karan@mf.uns.ac.rs (S.K.); stanislava.nikolic@mf.uns.ac.rs (S.N.); 015169@mf.uns.ac.rs (I.Ž.); 2 Clinic for Orthopedic Surgery and Traumatology, University Clinical Center of Vojvodina, 21137 Novi Sad, Serbia; 3 Center of Research Excellence in Nutrition and Metabolism, Institute for Medical Research, National Institute of Republic of Serbia, University of Belgrade, 11129 Belgrade, Serbia; ivana.sarac@imi.bg.ac.rs; 4 Center of Laboratory Medicine, University Clinical Center of Vojvodina, 21137 Novi Sad, Serbia

## 1. Text Corrections

There was an error in the original publication [1]. There were typing mistakes in Abstract and Results, Sections 3.4 and 3.5. 

In the Abstract and Section 3.5. (Serum Vitamin D Status), the first paragraph, incorrect serum vitamin D levels data in the fracture group, 40.0 (23.0–50.0) nmol/L instead of 23.0 (16.0–40.0) nmol/L, were included in the published version of the manuscript. 

In addition, there were errors in the Section 3.4. (The Intake of Vitamin D and Calcium through Food and Supplements), the second and the fifth paragraphs; there were typing mistakes related to the number and percentages of subjects who met the recommended dietary intakes of vitamin D and the percentages of subjects who did not meet the recommended dietary intakes of calcium. 

The corrected text appears below:

### 1.1. Abstract

Serum vitamin D levels were significantly lower in the fracture group (23.0 nmol/L vs. 76.0 nmol/L, *p* < 0.001).

### 1.2. Results. Section 3.4. The Intake of Vitamin D and Calcium through Food and Supplements, the Second Paragraph

In the case group, 95 subjects (90.5%) had vitamin D intake < 5 µg/day, while only 3 subjects (2.9%) had daily adequate intake according to the EFSA recommendations (ranging 17.4–30.2 µg/day), while in the control group, 15 subjects (14.3%) had an adequate vitamin D intake (ranging 15.2–43.3 µg/day), while 42 subjects (40.0%) and 41 subjects (39.0%) had vitamin D intake < 5 µg/day and 5–10 µg/day, respectively.

### 1.3. Results, Section 3.4. The Intake of Vitamin D and Calcium through Food and Supplements, the Fifth Paragraph

At the same time, 99 subjects (94.3%) and 80 subjects (76.2%), in the case and control group, respectively, did not meet the recommended dietary intakes by the IOM recommendations (1200 mg/day) (*p* = 0.001), while according to the EFSA recommendations (950 mg/day), 93 subjects (88.6%) and 53 subjects (50.5%), in the case and control group, respectively, did not meet the recommended dietary intakes *(p* < 0.001).

### 1.4. Results, Section 3.5. Serum Vitamin D Status, First Paragraph

The serum vitamin D, defined as total 25(OH)D serum levels, in the fracture group were statistically significantly lower compared to the control group: median (IQR), respectively, 23.0 (16.0–40.0) nmol/L vs. 76.0 (57.0–91.0) nmol/L (Mann-Whitney test *p* < 0.001) (Figure 4 and Supplementary Table S4). 

## 2. Errors in Table

In the original publication, there was also a mistake in Supplementary Table S3. as published (Table S3. Comparison of calcium and vitamin intake according to the EFSA and IOM recommendations between elderly subjects (>65 years) with and without fractures in the Vojvodina region, Serbia; Supplementary Material, at: https://www.mdpi.com/article/10.3390/nu16162702/s1 (accessed on 7 January 2026)). There were typing mistakes related to the number and percentages of subjects who met the recommended dietary intakes of vitamin D and the percentages of subjects who did not meet the recommended dietary intakes of calcium. The corrected Supplementary Table S3 appears below. **Supplementary Table S3.** Comparison of calcium and vitamin D intake according to the EFSA and IOM recommendations between elderly subjects (>65 years) with and without fractures in the Vojvodina region, Serbia.
**With Fractures (*n* = 105)****Controls (*n* = 105)**

**Median/*n*****(IQR)/(%)****Median/*n*****(IQR)/(%)*****p*****Calcium intake (mg/day)**536.7(420.4-688.9)945.0(671.7-1192.2)**<0.001****EFSA:****<0.001**Calcium intake < 950 mg/day *n* (%)93(88.6%)53(50.5%)Calcium intake 950–2500 mg/day *n* (%)12(11.4%)50(47.6%)Calcium intake ≥ 2500 mg/day *n* (%)0(0.0%)2(1.9%)**IOM:****0.001**Calcium intake < 1200 mg/day *n* (%)99(94.3%)80(76.2%)Calcium intake 1200–2000 mg/day *n* (%)6(5.7%)23(21.9%)Calcium intake ≥ 2000 mg/day *n* (%)0(0.0%)2(1.9%)**Vitamin D intake (µg/day)**1.4(0.9-2.7)5.8(3.3-8.6)**<0.001**Vitamin D intake < 5 µg/day *n* (%)95(90.5%)42(40.0%)**<0.001**Vitamin D intake 5–10 µg/day *n* (%)6(5.7%)41(39.0%)Vitamin D intake 10–15 µg/day *n* (%)1(1.0%)7(6.7%)Vitamin D intake 15–20 µg/day *n* (%)2(1.9%)4(3.8%)Vitamin D intake > 20 µg/day *n* (%)1(1.0%)11(10.4%)**EFSA:****0.005**Vitamin D intake < 15 µg/day *n* (%)102(97.1%)90(85.7%)Vitamin D intake ≥ 15 µg/day *n* (%)3(2.9%)15(14.3%)**IOM/Endocrine Society:****0.005**Vitamin D intake < 20 µg/day *n* (%)104(99.0%)94(89.5%)Vitamin D intake ≥ 20 µg/day *n* (%)1(1.0%)11(10.5%)EFSA = European Food Safety Authority, IOM = Institute of Medicine, IQR = interquartile range, *p* = statistical significance of difference (bolded values are statistically significant, *p* < 0.05).

The authors apologize for any inconvenience caused and state that the scientific conclusions are unaffected. This correction was approved by the Academic Editor. The original publication has also been updated.
