# Supplementary material for: Impact of Vitamin D Status and Nutrition on the Occurrence of Long Bone Fractures Due to Falls in Elderly Subjects in the Vojvodina Region of Serbia
Source: Nutrients. 2024 Aug 14;16(16):2702. doi: 10.3390/nu16162702 (PMC11356805; doi:10.3390/nu16162702)
Supplement: Supplementary file 1 [file nutrients-16-02702-s001.zip › nutrients-3144557-supplementary.pdf]

Supplementary material: Impact of vitamin D status and nutrition on the occurrence of long bone fractures due to falls in elderly subjects in the Vojvodina region of Serbia

Supplementary tables:

Supplementary Table S1. Comparison of energy intake and proportion of energy intake coming from different macronutrient categories according to the US dietary guidelines between elderly subjects (>65 years) with and without fractures in the Vojvodina region, Serbia

	**With fractures (n = 105)**		**Controls (n = 105)**		
	n	**(%)**	***n***	**(%)**	***p***
**Total energy intake:**					
• < 1600 kcal/day (F), < 2000 kcal/day (M)	92	(87.6%)	42	(40.0%)	**<0.001**
• ≥ 1600 kcal/day (F), ≥ 2000 kcal/day (M)	13	(12.4%)	63	(60.0%)	
**Percentage of energy intake coming from proteins:**					
• < 10%	24	(22.9%)	2	(1.9%)	**<0.001**
• 10–35%	81	(77.1%)	103	(98.1%)	
• >35%	0	(0.0%)	0	(0.0%)	
**Percentage of energy intake coming from fats:**					
• < 20%	0	(0.0%)	1	(1.0%)	**<0.001**
• 20–35%	40	(38.1%)	11	(10.5%)	
• >35%	65	(61.9%)	93	(88.6%)	
**Percentage of energy intake coming from carbohydrates:**					
• < 45%	37	(35.2%)	89	(84.8%)	**<0.001**
• 45–65%	67	(63.8%)	16	(15.2%)	
• >65%	1	(1.0%)	0	(0.0%)	

F = females, M = males, p = statistical significance of difference (bolded values are statistically significant, p<0.05).

Supplementary Table S2. Comparison of proportion of energy intake coming from different food groups between elderly subjects (>65 years) with and without fractures in the Vojvodina region, Serbia

Food groups' energy contribution	With fractures (*n* = 105)		Controls (*n* = 105)		
	**Median**	**(IQR)**	**Median**	**(IQR)**	***p***
Percentage of energy intake from milk and milk products (%Kcal/day)	7.1	(2.9-13.0)	12.8	(7.3-19.1)	**0.003**
Percentage of energy intake from eggs and egg products (%Kcal/day)	0.9	(0.0-3.8)	3.5	(0.9-7.3)	0.623
Percentage of energy intake from meat and meat products (%Kcal/day)	9.7	(5.6-17.7)	10.3	(4.1-18.1)	**<0.001**
Percentage of energy intake from fish, seafood, and related products (%Kcal/day)	0.0	(0.0-0.0)	5.5	(0.0-10.2)	0.238
Percentage of energy intake from edible fats, oil, and similar products (%Kcal/day)	12.6	(8.0-17.5)	14.9	(10.6-20.0)	0.467
Percentage of energy intake from grains and grain products (%Kcal/day)	35.0	(27.8-47.39	33.0	(24.7-38.5)	0.050
Percentage of energy intake from nuts, seeds, and related products (%Kcal/day)	1.7	(0.1-4.1)	1.2	(0.2-3.4)	0.998
Percentage of energy intake from vegetables and vegetable products (%Kcal/day)	5.4	(2.6-9.4)	4.0	(2.6-7.6)	0.119
Percentage of energy intake from fruit and fruit products (%Kcal/day)	3.3	(0.0-8.1)	3.1	(0.2-7.4)	**0.043**
Percentage of energy intake from sugar and sugar products (%Kcal/day)	5.0	(0.8-12.1)	1.2	(0.0-4.0)	**0.001**
Percentage of energy intake from alcoholic and non-alcoholic beverages (%Kcal/day)	2.7	(0.1-6.7)	1.9	(0.1-3.7)	**0.005**
Percentage of energy intake from miscellaneous food products (%Kcal/day)	0.0	(0.0-0.1)	0.1	(0.0-0.2)	**0.018**
Percentage of energy intake from special nutritional supplements (%Kcal/day)	0.0	(0.0-0.0)	0.0	(0.0-0.09	0.774

IQR = interquartile range, *p* = statistical significance of difference (bolded values are statistically significant, p<0.05).

Supplementary Table S3. Comparison of calcium and vitamin intake according to the EFSA and IOM recommendations between elderly subjects (>65 years) with and without fractures in the Vojvodina region, Serbia

	**With fractures (n = 105)**		**Controls (n = 105)**		
	**Median/*n***	**(IQR)/(%)**	**Median/*n***	**(IQR)/(%)**	***p***
**Calcium intake (mg/day)**	536.7	(420.4-688.9)	945.0	(671.7-1192.2)	**<0.001**
**EFSA:**					
• Calcium intake < 950 mg/day *n* (%)	93	(90.5%)	53	(40.0%)	**<0.001**
• Calcium intake 950-2500 mg/day *n* (%)	12	(5.7%)	50	(39.0%)	
• Calcium intake ≥ 2500 mg/day *n* (%)	0	(0.0%)	2	(3.8%)	
**IOM:**					
• Calcium intake < 1200 mg/day *n* (%)	93	(90.5%)	53	(40.0%)	**<0.001**
• Calcium intake 1200-2000 mg/day *n* (%)	12	(5.7%)	50	(39.0%)	
• Calcium intake ≥ 2000 mg/day *n* (%)	0	(0.0%)	2	(3.8%)	
**Vitamin D intake (μg/day)**	1.4	(0.9-2.7)	5.8	(3.3-8.6)	**<0.001**
• Vitamin D intake < 5 μg/day *n* (%)	95	(90.5%)	42	(40.0%)	
• Vitamin D intake 5-10 μg/day *n* (%)	6	(5.7%)	41	(39.0%)	
• Vitamin D intake 10-15 μg/day *n* (%)	1	(1.0%)	7	(6.7%)	**<0.001**
• Vitamin D intake 15-20 μg/day *n* (%)	2	(1.9%)	4	(3.8%)	
• Vitamin D intake > 20 μg/day *n* (%)	1	(1.0%)	11	(10.4%)	
**EFSA:**					
• Vitamin D intake < 15 μg/day *n* (%)	102	(79.1%)	90	(85.7%)	**<0.001**
• Vitamin D intake ≥ 15 μg/day *n* (%)	3	(2.9%)	15	(14.3%)	
**IOM/Endocrine Society:**					
• Vitamin D intake < 20 μg/day *n* (%)	104	(99.0%)	94	(89.6%)	**<0.001**
• Vitamin D intake ≥ 20 μg/day *n* (%)	1	(1.0%)	11	(10.4%)	

EFSA = European Food Safety Authority, IOM = Institute of Medicine, IQR = interquartile range, p = statistical significance of difference (bolded values are statistically significant, $p < 0.05$).

Supplementary Table S4. Comparison of serum vitamin D levels according to the IOF, Endocrine Society, American Geriatrics Society, EFSA, and IOM recommendations between elderly subjects (>65 years) with and without fractures in the Vojvodina region, Serbia

	**With fractures (*n* = 105)**		**Controls (*n* = 105)**		
	**Median/*n***	**(IQR)/(%)**	**Median/*n***	**(IQR)/(%)**	***p***
**Serum 25(OH)D levels (mmol/l)**	23.0	(16.0-40.0)	76.0	(57.0-91.0)	**<0.001**
**IOF/Scientific Advisory Committee on Nutrition (UK)/Endocrine Society, American Geriatrics Society (USA):**					
• Serum 25(OH)D levels < 25 mmol/l *n* (%)	55	(52.4%)	2	(1.9%)	
• Serum 25(OH)D levels 25-50 mmol/l *n* (%)	43	(41.0%)	12	(11.4%)	**<0.001**
• Serum 25(OH)D levels 50-75 mmol/l *n* (%)	7	(6.7%)	38	(36.2%)	
• Serum 25(OH)D levels ≥ 75 mmol/l *n* (%)	0	(0.0%)	53	(50.5%)	
**IOM/EFSA:**					
• Serum 25(OH)D levels < 30 mmol/l *n* (%)	63	(60.0%)	2	(1.9%)	**<0.001**
• Serum 25(OH)D levels 30-50 mmol/l *n* (%)	35	(33.3%)	12	(11.4%)	
• Serum 25(OH)D levels ≥ 50 mmol/l *n* (%)	7	(6.7%)	91	(86.7%)	

IOF = International Osteoporosis Foundation, EFSA = European Food Safety Authority, IOM = Institute of Medicine, IQR = interquartile range, *p* = statistical significance of difference (bolded values are statistically significant, p<0.05).

Supplementary Table S5. Logistic regression – univariate models for all candidate variables for predicting the risk for fractures

Univariate models	Candidate covariate	B	Exponent (B)/Odds ratio	95% CI for Exponent (B)	Predictor significance *p*	Model Nagelkerke R^2	Model significance *p*
1.	Sex (female)	0.000	1.000	(0.530-1.887)	1.000	1.000	1.000
	Constant	0.000	1.000		1.000		
2.	Age (years)	0.063	1.065	(1.014-1.119)	**0.012**	0.041	**0.010**
	Constant	-4.630	0.010		**0.012**		
3.	BMI (kg/m²)	-0.082	0.921	(0.850-0.999)	**0.046**	0.026	**0.043**
	Constant	2.158	8.658		**0.048**		
4.	Physical activity level (1-5)	-1.318	0.268	(0.182-0.394)	**<0.001**	0.377	**<0.001**
	Constant	4.567	96.248		**<0.001**		
5.	Smoking status (1-5)	0.429	1.536	(1.271-1.855)	**<0.001**	0.132	**<0.001**
	Constant	-0.708	0.493		**0.001**		
6.	Season (late autumn/early winter)	0.291	1.338	(0.759-2.357)	0.314	0.006	0.313
	Constant	-0.104	0.901		0.547		
7.	Protein dietary intake (g/day)	-0.059	0.943	(0.927-0.959)	**<0.001**	0.378	**<0.001**
	Constant	3.406	30.147		**<0.001**		
8.	Protein dietary intake (g/kg/day)	-3.789	0.023	(0.007-0.072)	**<0.001**	0.344	**<0.001**
	Constant	3.068	21.493		**<0.001**		
9.	Fat dietary intake (g/day)	-0.050	0.951	(0.936-0.966)	**<0.001**	0.337	**<0.001**
	Constant	3.569	35.478		**<0.001**		
10.	Fiber dietary intake (g/day)	-0.083	0.920	(0.871-0.971)	**0.003**	0.069	**<0.001**
	Constant	1.134	3.108		**0.004**		
11.	Vitamin D dietary intake (µg/day)	-0.297	0.743	(0.663-0.833)	**<0.001**	0.300	**<0.001**
	Constant	1.211	3.357		**<0.001**		
12.	Calcium dietary intake (mg/day)	-0.003	0.997	(0.996-0.998)	**<0.001**	0.299	**<0.001**
	Constant	2.477	11.901		**<0.001**		
13.	Milk and dairy product dietary intakes (Kcal/day)	-0.006	0.994	(0.992-0.996)	**<0.001**	0.192	**<0.001**
	Constant	1.021	2.776		**<0.001**		
14.	Egg and egg product dietary intakes (Kcal/day)	-0.011	0.989	(0.984-0.994)	**<0.001**	0.153	**<0.001**
	Constant	0.609	1.839		**0.001**		
15.	Fish and seafood dietary intakes (Kcal/day)	-0.018	0.982	(0.976-0.988)	**<0.001**	0.374	**<0.001**
	Constant	0.787	2.197		**<0.001**		
16.	Edible fats and oils dietary intakes (Kcal/day)	-0.006	0.994	(0.992-0.997)	**<0.001**	0.154	**<0.001**
	Constant	1.317	3.733		**<0.001**		
17.	Sugar and sweets dietary intakes (Kcal/day)	0.007	1.007	(1.003-1.010)	**<0.001**	0.116	**<0.001**
	Constant	-0.502	0.605		**0.007**		
18.	Alcoholic and non-alcoholic beverages (Kcal/day)	0.007	1.007	(1.002-1.012)	**0.007**	0.056	**0.003**
	Constant	-0.331	0.718		0.067		
19.	Serum 25(OH)D levels (nmol/L)	-0.123	0.884	(0.854-0.915)	**<0.001**	0.755	**<0.001**
	Constant	5.951	383.986		**<0.001**		
20.	Vitamin D supplements intake (yes)	-0.862	0.422	(0.126-1.417)	0.163	0.013	0.147
	Constant	0.051	1.052		0.722		

BMI = body mass index, B = logistic regression coefficient, CI = confidence interval, R^2 = coefficient of determination, *p* = statistical significance (bolded values are statistically significant, p<0.05).

Supplementary figure

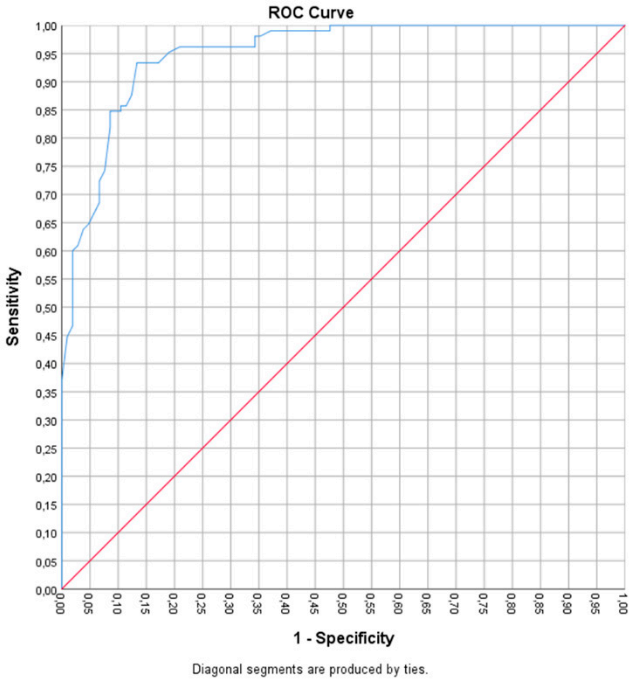

Supplementary Figure S1. ROC curve analysis for sensitivity and specificity of serum 25(OH)D cut-off value of 50.5 nmol/L for predicting the fracture risk among examined elderly subjects.
